# Optimising storage conditions and processing of sheep urine for nitrogen cycle and gaseous emission measurements from urine patches

**DOI:** 10.1038/s41598-021-91498-4

**Published:** 2021-06-09

**Authors:** Alice F. Charteris, Karina A. Marsden, Jess R. Evans, Harry A. Barrat, Nadine Loick, Davey L. Jones, David R. Chadwick, Laura M. Cárdenas

**Affiliations:** 1 Sustainable Agriculture Sciences, Rothamsted Research, North Wyke, Okehampton, EX20 2SB DEV UK; 2 School of Natural Sciences, Bangor University, Bangor, LL57 2UW GWN UK; 3 Faculty of Veterinary and Agricultural Sciences, University of Melbourne, Parkville, VIC 3010 Australia; 4 Rothamsted Research, Harpenden, Hertfordshire, AL5 2JQ UK; 5 SoilsWest, UWA School of Agriculture and Environment, The University of Western Australia, Perth, WA 6009 Australia

**Keywords:** Climate sciences, Environmental sciences

## Abstract

In grazing systems, urine patches deposited by livestock are hotspots of nutrient cycling and the most important source of nitrous oxide (N_2_O) emissions. Studies of the effects of urine deposition, including, for example, the determination of country-specific N_2_O emission factors, require natural urine for use in experiments and face challenges obtaining urine of the same composition, but of differing concentrations. Yet, few studies have explored the importance of storage conditions and processing of ruminant urine for use in subsequent gaseous emission experiments. We conducted three experiments with sheep urine to determine optimal storage conditions and whether partial freeze-drying could be used to concentrate the urine, while maintaining the constituent profile and the subsequent urine-derived gaseous emission response once applied to soil. We concluded that filtering of urine prior to storage, and storage at − 20 °C best maintains the nitrogen-containing constituent profile of sheep urine samples. In addition, based on the 14 urine chemical components determined in this study, partial lyophilisation of sheep urine to a concentrate represents a suitable approach to maintain the constituent profile at a higher overall concentration and does not alter sheep urine-derived soil gaseous emissions.

## Introduction

Urine patches represent hotspots of nutrient input in grazing systems^1^. Within patches, excessive soil nitrogen (N) concentrations increase the likelihood of N loss from the soil, occurring via ammonia (NH_3_) volatilisation, nitrate (NO_3_^-^) leaching and gaseous nitric oxide (NO), nitrous oxide (N_2_O) or nitrogen (N_2_) losses (mainly released through nitrification and denitrification^2^). These losses also represent inefficient recycling of a valuable nutrient. Hence, urine patch N dynamics form the basis for a large body of research, especially in countries where pasture based ruminant production represents an important component of their total N_2_O emissions (e.g. New Zealand^3^ and Ireland^4^).

Due to the difficulties in obtaining (e.g. animal welfare concerns, health and safety), handling (potential health risks associated with biological materials, i.e. urine), storing (ensuring composition remains unchanged) and controlling the composition of natural urine, artificial urine is commonly used in controlled experimentation on N dynamics e.g.^5–8^. Such artificial urine solutions are more straightforward to generate and handle; can be manipulated to the desired concentrations (e.g. N) and constituent balances; and are easily replicated ^9–12^. However, the importance of minor urine constituents, which may not all be included in synthetic mixtures, remains uncertain. Gardiner et al.^13^ found that increasing the concentration of five minor components (allantoin, creatinine, creatine, uric acid and [hypo]xanthine) did not affect urine patch N dynamics, including N_2_O emissions, while increased urine hippuric acid content has been found to inhibit soil N_2_O fluxes in some studies e.g.^14^, but not others e.g.^15^. Differing concentrations of minor constituents was posited to be the reason for lower sheep urine N_2_O emission factors than those from cattle urine, independently of the volume and N concentration of the urine^16^, but other factors (e.g. potassium ion [K^+^] concentration or urine pH), for which insufficient data for meta-analysis were provided, could also have an effect. Standard synthetic urine cannot exactly mimic real urine (i.e. fails to capture the full complement of natural compounds/elements and may be missing minor biologically active soil microbial enhancers or inhibitors), and subsequent experimental results can differ from those of real urine e.g.^5,9,11^. Experiments involving natural urine are therefore advocated^17^ and are sometimes necessary, even if only to confirm that results from synthetic mixtures adequately represent those from natural urine.

Advice regarding urine collection is available e.g.^18,19^. Yet, few studies, outside the medical and veterinary fields in which urine samples need to be preserved for analysis, rather than further use/experimentation^20–22^, have investigated the best preparation and storage methods for animal urine for use in experiments. A common method of urine preservation is by acidification with sulphuric (H_2_SO_4_) or hydrochloric (HCl) acid to prevent bacterial constituent degradation and NH_3_ volatilisation e.g.^23,24^. However, this is unsuitable for urine to be used in further experiments (e.g. urine-amended soil emission assessments) as it alters its composition and pH. Generally accepted procedures include refrigeration (< 4 °C) for short term storage (up to *ca.* 48 h; e.g. Traum et al.^25^ demonstrated that refrigerator storage was suitable for human urine samples for 24 h); and freezing (< − 20 °C) for longer term storage. However, in our experience, detailed descriptions of urine preparation methods (or even sometimes basic analysis of the urine itself^16^) are often not included in publications and little quantitative information is available to support these approaches.

Another common dilemma in studies involving animal urine is how best to achieve a representative natural urine treatment for use in experiments. Unless the variation between events/animals is being investigated, it is often desirable to pool urine from more than one urination event and animal to obtain an average urine treatment and ensure unplanned/additional treatment differences are not introduced to the experiment by differing urine compositions e.g.^26–28^. The concentrations of constituents in natural urine cannot be controlled, however, and pooled samples by their nature, converge to the average urine concentration, making it difficult to achieve a number of concentration levels spanning the range of naturally occurring concentrations. In studies investigating the effect of urine concentration on emissions of the powerful greenhouse gas, N_2_O, this is commonly circumvented either by using synthetic urine at a range of concentrations e.g.^10^, by dilution of natural urine (resulting in a low-end range) e.g.^29,30^ or by urea-N addition to natural urine e.g.^26,31,32^. These solutions all have drawbacks, viz., (i) standard synthetic urine mixtures may not sufficiently/accurately represent natural urine, (ii) dilution of a pooled natural urine sample to give various concentration levels excludes high urine-N concentration treatments (and N_2_O emissions do not always increase linearly with N application or urine concentration)^33,34^, and iii) urea-N addition enables higher urine N concentrations to be tested, but alters the constituent balance of the natural urine reducing the relative concentration of other compounds (e.g. purine derivatives) if they are not also added. Indeed, the use of real urine is recommended for the investigation of urine patch N_2_O emissions and determination of emission factors^17^, the accuracy of which are vital for realistic estimation of regional and national N_2_O emissions.

Herein, we present the results of a study to optimise urine sample storage for subsequent urine patch N-cycle and gaseous emission (carbon dioxide [CO_2_], N_2_O and NO) experiments. We hypothesised that urine filtration to remove suspended solids (e.g. any faecal contamination) and microbial contaminants responsible for urea hydrolysis, followed by frozen storage would best maintain chemical composition. We also tested whether a functionally normal (in terms of gas emissions) pooled natural urine sample of high concentration could be obtained by water removal, which, if done carefully, could also maintain the balance of other constituents in the urine sample. Water removal could be achieved via heating and evaporation (e.g. rotary evaporation) or via freeze-drying. Since heating encourages urea hydrolysis and NH_3_ volatilisation^2^, freeze-drying was deemed the preferable approach to test. Therefore, we hypothesised that freeze-drying would not affect urine composition or function in terms of its effect on gaseous emissions from urine-amended soil.

## Results

### Urine storage tests

**Figure 1:**
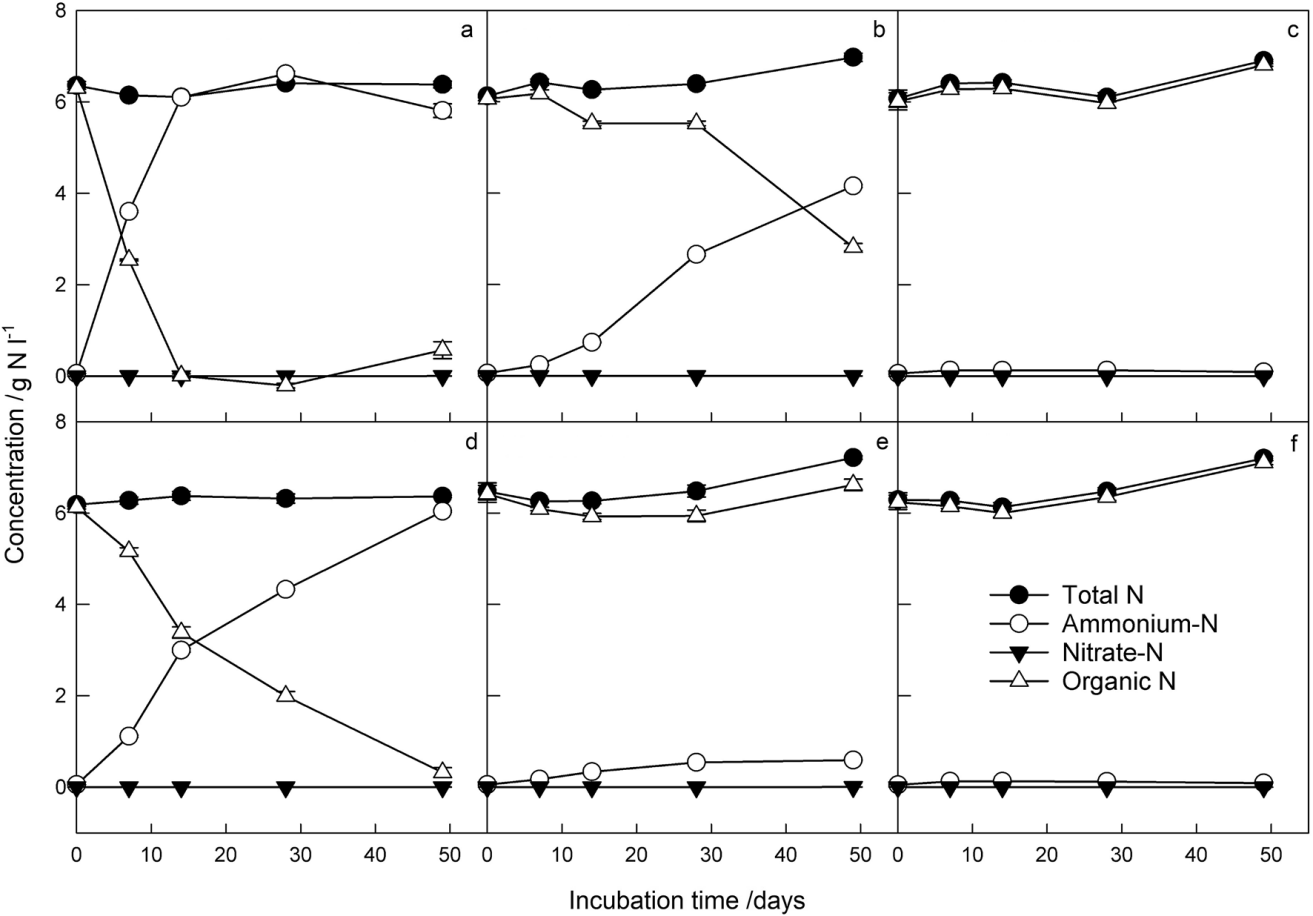
Urine N concentrations of unfiltered (**a**–**c**) and filtered (**d**–**f**) samples stored at room temperature (**a** and **d**), in a refrigerator (8 °C; **b** and **e**) or in a freezer (-20 °C; **c** and **f**) for up to 49 days. Error bars are ± standard errors of the means (SEMs; n = 3), average confidence intervals (CIs) for the full fixed model (filtering*temperature*time) were as follows: total N mean ± 0.170; NH_4_^+^-N mean ± 1.05; NO_3_^–^N mean ± 1.28; total organic N mean ± 0.192.

The temporal changes in concentrations (g N l^−1^) of total N, ammonium N (NH_4_^+^-N), NO_3_^–^N and total organic N in urine in each storage treatment (filtered/unfiltered, at room temperature/refrigerated/frozen) are shown in Fig. 1. Comparison of the figure panels clearly shows that NH_4_^+^-N and total organic N were strongly affected by storage temperature, with NH_4_^+^-N concentrations increasing and total organic N concentrations decreasing over time at room temperature (Fig. 1a,d), but to a lesser extent when refrigerated (Fig. 1 b,e) or frozen (Fig. 1c,f). Filtering lessened these changes over time at room and refrigerator temperatures. Indeed, for NH_4_^+^-N and total organic N, all individual treatments, all two-way interactions and the three-way interaction were highly significant (*P* < 0.001; Supplementary Table 2).

Although differences were less clear for total N and NO_3_^–^N concentrations, the three-way interaction was also highly significant for these components (F_8,17_ = 6.04 and F_8,16_ = 6.33 for total N and LN[NO_3_^–^N], respectively, *P* < 0.001 for both) indicating that all treatment factors were important. Thus, while filtering alone was not found to significantly affect total N or NO_3_^–^N concentrations (F_1,12_ = 4.31 and 0.82, *P* = 0.060 and 0.382 for total N and LN[NO_3_^–^N] respectively), the effect of filtering was significantly different at different time points (filtering*time F_4,14_ = 4.58 and F_4,13_ = 15.94, *P* = 0.014 and < 0.001 for total N and LN[NO_3_^–^N], respectively). Storage temperature and time had highly significant effects on both total N and NO_3_^–^N concentrations individually (*P* < 0.001 for all; Supplementary Table 2) and in combination (temperature*time *P* < 0.001 for both; Supplementary Table 2). Higher total N concentrations occurred at lower temperatures (refrigerated and frozen) and later time points, while higher NO_3_^–^N concentrations occurred at higher temperatures and later time points for filtered samples while unfiltered samples were more erratic.

### Freeze-dried urine composition

Two separate experiments were conducted to assess the compositional and soil gaseous emission effects of freeze-drying and rehydrating sheep urine. Urine composition was assessed in both experiments by measuring the concentrations of total dissolved C and N, urea, NH_4_^+^-N, NO_3_^–^N, total free amino acids (TFAAs), sodium (Na^+^), K^+^ and calcium (Ca^2+^) cations, allantoin, creatinine, uric acid, hippuric acid and benzoic acid, before and after freeze-drying and rehydrating urine samples. Emissions of CO_2_ and N_2_O were assessed in the first freeze-drying experiment (FD-1) and NO in the second freeze-drying experiment (FD-2) from non-freeze-dried and freeze-dried urine samples applied to soil using the controlled and automated, flow-through Denitrification Incubation System (DENIS)^35^.

**Figure 2:**
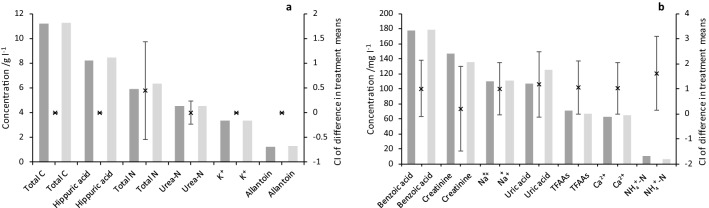
FD-1. Mean non-freeze-dried (darker bars; N) and freeze-dried (lighter bars; F) urine composition data: (**a**) Constituents with mean concentrations over 1 g l^−1^, (**b**) Constituents with mean concentrations under 200 mg l^−1^. Error bars show the CI of the difference between the treatment means (N *vs.* F; n = 6) and are centred on the difference between the means. (Note that 0 mg NO_3_^–^N l^−1^ was recorded in all samples and is not shown).

**Figure 3:**
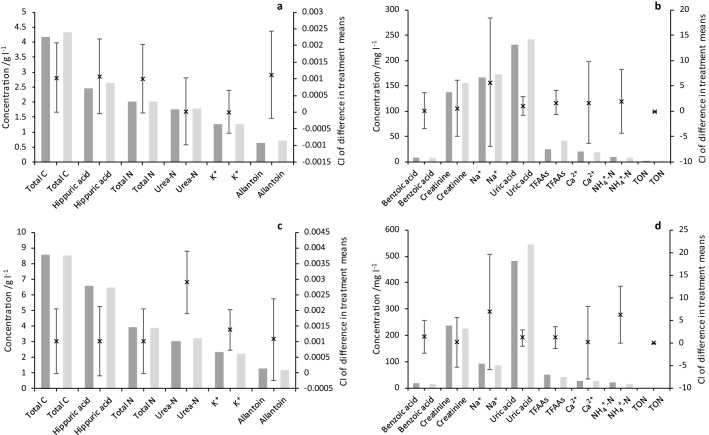
FD-2. Mean non-freeze-dried (darker bars; N) and freeze-dried (lighter bars; F) urine composition data: (**a**–**b**) Low concentration mixtures (LM), (**c**)–(**d**) High concentration mixtures (HM; Supplementary Table 1), (**a**) and (**c**) Constituents with mean concentrations over 1 g l^−1^, (**b**) and (**d**) Constituents with mean concentrations under 600 mg l^−1^. Error bars show the CI of the difference between the treatment means (N *vs.* F; n = 3) and are centred on the difference between the means.

The mean concentrations of the 14 constituents in the non-freeze-dried and freeze-dried urine samples in the two experiments are shown in Figs. 2 and 3. For the six different urine samples in FD-1, non-freeze-dried urine total carbon (C) concentrations ranged from 3.2 to 22.2 g C l^−1^ and total N concentrations ranged from 1.4 to 9.5 g N l^−1^. In general, constituent concentrations were consistent (i.e. samples with high/low total C and N concentrations had high/low concentrations of other constituents). Urine Na^+^ concentrations were an exception to this. In FD-2, six different urine mixtures were used to achieve two urine total N concentration levels (*ca.* 2 and 4 g N l^−1^) and results indicate this was approximately achieved. Although there was still some consistency in concentration across constituents, this was less clear than in FD-1. However, urine Na^+^ concentrations were again clearly independent of overall total C and N concentrations.

In FD-1, only the concentration of NH_4_^+^-N was significantly lower (transformed mean 10.2 mg N l^−1^ non-freeze-dried *vs.* 6.3 mg N l^−1^ freeze-dried; Fig. 2b; LN[NH_4_^+^-N] F_1,5_ = 9.75, *P* = 0.026; Supplementary Table 3) at the 5% significance level (*P* < 0.05) following freeze-drying and rehydration. For FD-2, as expected due to the experimental design, there was a highly significant difference between the two N concentrations levels (LN[Total N] F_1,4_ = 246.38, *P* < 0.001; Supplementary Table 3), and since the concentrations of other constituents were somewhat correlated, significant differences with concentration level were observed for most other constituents, with the exception of lower concentration constituents such as NH_4_^+^-N and TON-N, for TFAAs and for Na^+^, (the concentration of the latter of which was earlier observed to be independent of the concentrations of other constituents). In FD-2, only the concentration of urea-N was significantly higher (transformed mean 1.77 and 3.02 g N l^−1^ non-freeze-dried *vs.* 1.78 and 3.21 g N l^−1^ freeze-dried for the low and high concentration groups, respectively; Fig. 3a,c; SQRT[Urea-N] F_1,4_ = 13.25, *P* = 0.022; Supplementary Table 3) at the 5% level following freeze-drying and rehydration. There was also a significant interaction at the 5% significance level between freeze-drying and concentration (suggesting that concentration affected how the samples reacted to freeze-drying) for urea-N and K^+^ (SQRT[Urea-N] and SQRT[K^+^] F_1,4_ = 9.34 and 9.37, respectively with, *P* = 0.038 in both cases; Supplementary Table 3).

### Freeze-dried urine gas emissions

**Figure 4:**
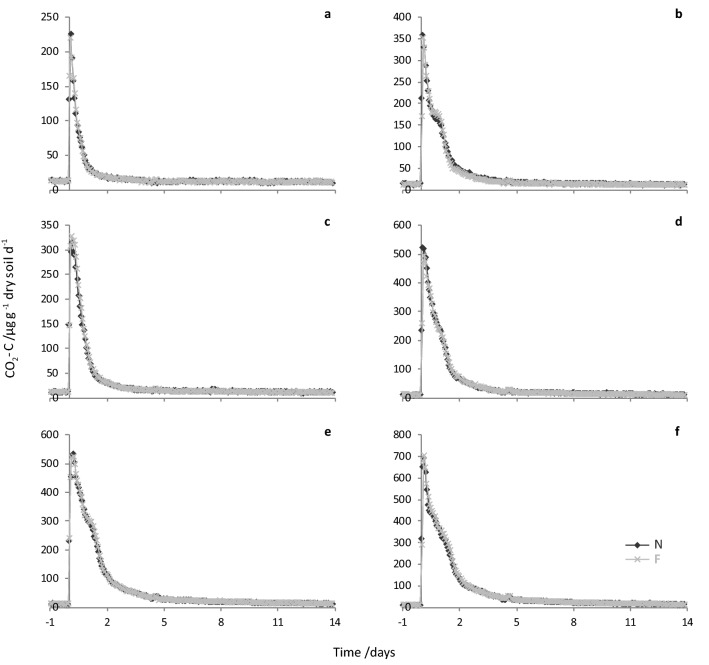
Graphs of paired CO_2_-C fluxes (ug g^−1^ dry soil d^−1^) from soil amended with non-freeze-dried (N; dark grey diamonds) or freeze-dried and rehydrated (F; light grey crosses) sheep urine samples in FD-1: (**a**) Sample ID 1, (**b**) 2, (**c**) 3, (**d**) 4, (**e**) 5, (**f**) 6.

**Figure 5:**
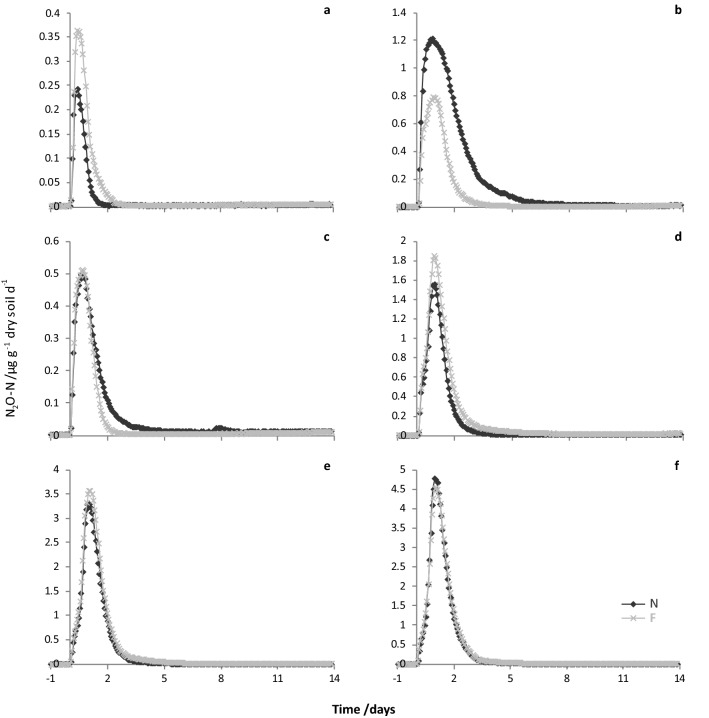
Graphs of paired N_2_O-N fluxes (ug g^−1^ dry soil d^−1^) from soil amended with non-freeze-dried (N; dark grey diamonds) or freeze-dried and rehydrated (F; light grey crosses) sheep urine samples in FD-1: (**a**) Sample ID 1, (**b**) 2, (**c**) 3, (**d**) 4, (**e**) 5, (**f**) 6.

**Figure 6:**
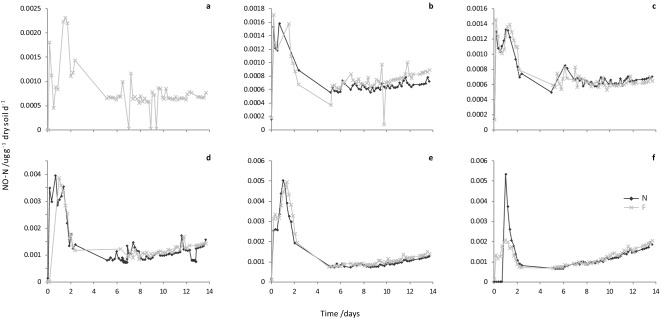
Graphs of paired NO-N fluxes (ug g^−1^ dry soil d^−1^) from soil amended with non-freeze-dried (N; dark grey diamonds) or freeze-dried and rehydrated (F; light grey crosses) sheep urine samples in FD-2: (**a**) LM1, (**b**) LM2, (**c**) LM3, (**d**) HM4, (**e**) HM5, (**f**) HM6. Gaps in data points (particularly between 2 and 6 days) result from instrument issues (i.e. no data was recorded during this time) in addition, a system error in the automated DENIS resulted in no data being recorded for LM1-N and only data for LM1-F is shown.

Emissions of CO_2_-C, N_2_O-N and NO-N (µg g^−1^ dry soil d^−1^) from soil amended with non-freeze-dried or freeze-dried sheep urine (pairs) are shown in Figs. 4, 5, 6. Maximum CO_2_-C fluxes ranged from 220 to 705 µg g^−1^ dry soil d^−1^ (1-F and 6-F, respectively), with higher total C and N concentration urine samples giving higher peak fluxes. Fluxes of CO_2_-C from the paired non-freeze-dried and freeze-dried sheep urine samples were similar, both in terms of the pattern and the magnitude of fluxes (emission peaks almost exactly overlay; Fig. 4). Accordingly, cumulative CO_2_-C emissions (µg g^−1^ dry soil; Table 1) were not significantly different between the non-freeze-dried and freeze-dried pairs (LN[CO_2_-C] F_1,5_ = 0.04, *P* = 0.858).

**Table 1:** Cumulative 14-day CO_2_-C, N_2_O-N and NO-N emissions (µg g^−1^ dry soil) from ‘paired’ soil units amended with non-freeze-dried (N) or freeze-dried (F) sheep urine. Urine samples are numbered and samples in FD-2 are identified by ‘LM’ for ‘low mixture’ or ‘HM’ for ‘high mixture’.

Sample ID	CO_2_–C	N_2_O–N	Sample ID	NO–N
	µg g^−1^ dry soil			µg g^−1^ dry soil
1-N	102	0.184	LM1-N	–
1-F	103	0.351	LM1-F	0.0117
2-N	323	2.79	LM2-N	0.0104
2-F	314	1.15	LM2-F	0.0106
3-N	252	0.793	LM3-N	0.0098
3-F	262	0.603	LM3-F	0.0100
4-N	585	1.82	HM4-N	0.0182
4-F	546	2.53	HM4-F	0.0183
5-N	770	4.05	HM5-N	0.0185
5-F	773	4.75	HM5-F	0.0202
6-N	960	5.61	HM6-N	0.0156
6-F	981	5.77	HM6-F	0.0157

Maximum N_2_O-N fluxes ranged from 0.24 to 4.8 µg g^−1^ dry soil d^−1^ (1-N and 6-N, respectively) and peak emissions again varied with urine total C and N concentrations. The patterns and magnitudes of N_2_O-N fluxes from the paired non-freeze-dried and freeze-dried sheep urine samples were not quite as consistent as for CO_2_-C, but there was still considerable agreement between the paired samples (Fig. 5). Cumulative N_2_O-N emissions (µg g^−1^ dry soil; Table 1) were also not significantly different between the non-freeze-dried and freeze-dried pairs (SQRT[N_2_O-N] F_1,5_ = 0.02, *P* = 0.894).

Maximum NO-N fluxes ranged from 1.3 × 10^–3^ to 5.4 × 10^–3^ µg g^−1^ dry soil d^−1^ (LM3-N and HM6-N; Fig. 6) and peak emissions were higher from the high concentration (HM) samples. Unfortunately, an unusually high number of sampling line restrictions/blockages (resulting in very low measured concentrations, followed by a peak in concentrations as a result of the build-up release) and instrument problems (e.g. data loss during days 2–6) affected the NO-N flux data from this experiment. However, this affected all experimental units in the same manner so does not compromise comparison of NO-N emissions from non-freeze-dried and freeze-dried pairs. Once again, although not as close as CO_2_-C fluxes, hourly NO-N fluxes from the paired non-freeze-dried and freeze-dried sheep urine samples compare well (Fig. 6). Cumulative NO-N emissions (µg g^−1^ dry soil; Table 1) were significantly different between the two concentrations levels (SQRT[NO-N] F_1,3_ = 34.23, *P* = 0.010), but not between the non-freeze-dried and freeze-dried pairs (SQRT[NO-N] F_1,3_ = 1.99, *P* = 0.253) and concentration did not affect the difference between the pairs (SQRT[NO-N] F_1,3_ = 0.49, *P* = 0.535).

## Discussion

### Urine preparation and storage conditions

The adequate preparation and storage of ruminant urine is an important experimental step to consider in the design and execution of experiments linked to the fate of urine-N once deposited to soil/the pasture environment, but very little information is available in the literature. Urine preservation by acidification e.g.^23,24^ is not appropriate for experimental use/application as it alters the pH and chemical composition of the urine. We assessed the effect of filtration and urine storage temperature to assess the best method of urine storage prior to experimental applications to avoid losses of urine N and changes in the urine N chemical profile.

Storing the urine at room temperature resulted in rapid urea hydrolysis (assessed by the decline in total organic N and increased urine NH_4_^+^-N concentrations) in both the filtered and particularly the unfiltered samples, suggesting this is an inappropriate method of storing urine. Refrigeration slowed urea hydrolysis in the unfiltered samples compared with storing at room temperature, but still resulted in changes over the incubation period. Filtering the urine slowed down the rates of urea hydrolysis in the refrigerated samples. It is now well established that filtering to pass 0.2 µm or 0.45 µm does not completely remove all microbial contaminants and cannot render a sample sterile^36^. Freezing to − 20 °C reduced hydrolysis even further, but still resulted in statistically significant N constituent concentration changes over time. Storage well below − 20 °C may be required to reduce this further – metabolic activity has been observed in hypersaline brine pockets in sea ice down to − 32°C^37,38^.

Overall, although no preparation and storage combination tested here could completely maintain the urine N profile over time, freezing (− 20 °C) represented the best option for longer-term storage (> 1 d). This has also been found in studies with human urine e.g.^39^. Storage tests by Laparre et al.^40^ with bovine urine similarly concluded that long-term storage at − 20 °C did not alter the abundance of most compounds, but that for some sensitive metabolites, freezing at − 80 °C was better to maintain urine composition, particularly over longer periods. The effect of faster freezing, for example in dry ice or with liquid N_2_ should also be investigated. In addition, while filtering (0.45 µm) did not appear to make a difference for frozen storage (Fig. 1), and statistically significant interactions for all N constituents confirmed that the effect of filtering differed at different temperatures, filtering was, on average, better for maintaining urine NH_4_^+^-N and total organic N concentrations. Filtering would certainly help to maintain the urine N composition between collection and frozen storage. Moreover, if defrosted urine behaves in the same way as fresh urine, pre-storage filtration could be useful to maintain the urine composition while defrosting and until use (in this study, urine samples were defrosted slowly, and we did not compare with rapid defrosting in warm water). We therefore recommend filtering and freezing urine as soon as possible after collection as the optimal way to avoid urine-urea hydrolysis and preserve the urine N composition profile for longer periods prior to experimental use. This aligns with recommendations for water samples e.g.^41^. Refrigerator storage should only be considered as a short-term solution (< 48 h), and filtering would also be recommended in this situation. Although no tests were conducted to specifically investigate filtration methods, the large effect of temperature on urea hydrolysis observed here, indicates that keeping urine chilled on ice during any preparation steps (e.g. filtration) would minimise this. In addition, in this study 0.45 µm filters (combined with vacuum filtration) were used, which may be impractical for filtering larger volumes of urine and further investigation of optimal filtration techniques (e.g. tangential flow ultrafiltration) and equipment is required (e.g. range of filter pore sizes – 0.2 µm filters may be better, or 100 kDa ultrafiltration could be used to remove urease, for example). Finally, collection and storage of urine in tightly sealed containers (with a small remaining headspace) may help to reduce NH_3_ volatilisation, although care should be taken as freezing tightly sealed containers of liquid with little room to expand can result in container rupture.

### Freeze-dried urine composition

The range of urine concentrations used in FD-1 enabled examination of the effects of freeze-drying across a concentration range for all constituents. The independent variation of urinary Na^+^ concentrations compared with other urine constituents could be due to a known physiological variation between individual sheep in the proportion of Na^+^ intake excreted in urine versus faeces^42^. This proportion also changes with Na^+^ intake, with an increasing percentage of Na^+^ intake excreted in urine with increasing intake^42^. Thus, urine samples with a low Na^+^ concentration could result from both a greater propensity for faecal Na^+^ excretion by that sheep and a lower Na^+^ intake. The higher urinary Na^+^ concentrations observed (up to *ca.* 550 mg Na^+^ l^−1^ in sample 3.2) indicate a high Na^+^ intake at which a large percentage of the Na^+^ was excreted in urine^42^, or possibly some contamination of the urine sample with faeces, although this was minimised through use of the muslin screen, continuous monitoring and immediate collection of urine samples.

Across the two experiments investigating the concentration of 14 important urine constituents, only NH_4_^+^-N in the FD-1 and urea-N in FD-2 were significantly different, and at the 5% level only between the non-freeze-dried and freeze-dried urine pairs. Urea is generally the major N-containing component of urine (up to 90% urine N), while NH_4_^+^ usually represents less than 1% of total urine N in fresh samples^34,43,44^. Urinary urea is readily hydrolysed (with a strong dependence on temperature) to NH_4_^+^, however, by urease enzymes which are ubiquitous in the environment and in animal faeces^45,46^. In FD-1, urea comprised between 50 and 81% total N, while NH_4_^+^ constituted less than 0.4%. In FD-2, urea represented upwards of 71% total N, while NH_4_^+^ was less than 1%. The reasons for the significant differences observed are unknown, but since only one constituent in each experiment (and a different one) was significantly different between the non-freeze-dried and freeze-dried pairs, this could result from small experimental or analytical errors, rather than the lyophilisation process. For urea, this rationale is supported by the numerically very small increase in urea concentrations (6% for the high concentration level and 0.7% for the low concentration level) following freeze-drying, and that the expected effect of lyophilisation would be a decrease in urea concentrations, rather than an increase. For NH_4_^+^-N, the difference was larger, but the concentration of urine in the sample was much lower. Urine was not diluted for either the urea or NH_4_^+^-N analyses, however, and rehydration errors would be expected to affect all constituents, so it is difficult to suggest any specific reason(s) for the differences.

Replication of concentration levels in FD-2 allowed statistical confirmation that freeze-drying effects were not dependent on concentration. Results indicated that this was the case, except perhaps for urea-N and K^+^. For urea-N, high concentration samples were consistently higher in urea-N with freeze-drying, while low concentration samples were not, which may explain the statistically significant interaction. Similarly, for K^+^, high concentration samples were consistently lower in K^+^ with freeze-drying, while low concentration samples were not. The reason for this is unknown but could result if lyophilisation affects these constituents proportionally (rather than additively) and the greater change can only be detected for the higher concentration samples. Additional experiments would be required to investigate this further.

Overall, these results suggest that partial freeze-drying can be used to concentrate sheep urine without affecting its composition. Freeze-drying (to dryness) has also been shown to be effective for preservation and analysis of human urine samples in the literature, for example: i) analysis of lyophilised urine headspace volatile organic compounds by solid phase micro-extraction—gas chromatography—mass spectrometry^47^; and ii) for very long term storage of human urine samples for later analysis of DNA^48^.

### Freeze-dried urine gas emissions

Importantly, there were no significant differences in the emissions of CO_2_-C, N_2_O-N and NO-N from urine-amended soils between the non-freeze-dried and freeze-dried urine sample pairs indicating that freeze-drying of sheep urine to concentrate it for use in gas emission monitoring experiments is a suitable method. However, it should be noted that we did not measure the emissions of all potentially important gases, for example, NH_3_ and methane (CH_4_). It is also difficult to assess whether differences are smaller between the non-freeze-dried and freeze-dried urine sample pairs than with artificial sheep urine as this has not been tested in a comparable experiment using sieved and repacked soils. Since differing experimental results from those of real urine have been recorded with synthetic urine mixtures e.g.^5,9,11^, freeze-drying urine to generate a mixed sample at various concentration levels may have advantages over corresponding artificial mixtures in terms of assessing urine-derived soil gas emissions. In addition, this approach may be suitable for investigating the effect of urine N concentration on N_2_O emissions for the development of country specific N_2_O emission factors, for which the use of natural urine under field conditions has been recommended^17^, although recently, artificial urine has been used^49^.

## Conclusions

This study provides valuable information not currently available in the literature regarding the preparation and storage of sheep urine samples for subsequent urine patch N-cycle and gaseous emission (CO_2_, N_2_O and NO) experiments. In agreement with our hypothesis, filtering of urine prior to storage and storage at − 20 °C best maintains the N constituent profile of sheep urine samples and can therefore be recommended for the preservation of urine for use in urine-N related experiments. In addition, while some statistically significant differences were observed between the composition of non-freeze-dried and freeze-dried urine samples, differences were small in the case of urea, and not consistent, suggesting that with careful handling, freeze-drying sheep urine to concentrate it is a suitable approach to maintain the constituent profile at a higher overall concentration. It should be noted this is based on the 14 components determined in this work; other minor constituents not investigated here may be sensitive to lyophilisation. These potential undetermined differences are not important in terms of sheep urine-derived soil gas emissions, however, as freeze-drying was not found to affect emissions. Freeze-drying sheep urine to concentrate it is therefore suitable for use in experiments investigating urine-derived soil gas emissions and indeed, may have advantages over other approaches such as artificial urine mixtures.

## Methods

### Urine collection

Sheep urine was collected from six Welsh Mountain ewes (*Ovis aries*) using urine collection pens (described in detail in Marsden et al.^44,50^; approved by Bangor University’s College of Natural Sciences Ethics Committee; code: CNS2016DC01) with slatted plastic flooring (designed for sheep; Rimco Ltd., Yorkshire, UK) lying over large, plastic collection trays. Urine collection was performed in accordance with relevant guidelines and regulations. The flooring and trays were separated by a muslin-lined mesh screen to reduce faeces, hoof debris, refused feed, wool or other contaminants from entering the urine collection trays. The collection pens were set up at the Henfaes Research Station, Abergwyngregyn, North Wales (53°13′13’’N, 4°0′34’’W) on a semi-improved, extensively managed 11.5 ha pasture 240–340 m above sea level (a.s.l.). The sheep were provided with water and forage freshly cut from the field site (mainly U4 and MG6 British NVC classified grasses: *Festuca ovina*, *Agrostis capillaris* and *Galium saxatile*; and *Lolium perenne* and *Cynosurus cristatus*, respectively)^51^. Urine from each urination event was collected from the trays individually, volumes recorded, and the samples stored in sealed bottles in cooler boxes prior to same-day processing and/or storage.

### Urine storage tests

A mixed subsample of the collected urine was used to determine the best method of urine storage to preserve composition. Unfiltered and filtered (0.45 µm, vacuum filtration) subsamples (18 experimental units in total) were stored at ambient laboratory temperature (room temperature), refrigerated (8 °C) or frozen (− 20 °C) and analysed for total N, NH_4_^+^-N and NO_3_^–^N on the day of collection and after 7, 14, 28 and 49 days. Total organic N was estimated by deducting inorganic N (NH_4_^+^-N and NO_3_^–^N) from total N.

Following dilution (1000-fold) with ultrapure water, total urine N was determined using a Multi N/C 2100S analyser (AnalytikJena, Jena, Germany). Urine NH_4_^+^-N (urine diluted tenfold with ultrapure water) and NO_3_^–^N (undiluted urine) were determined by colorimetric reactions and spectrophotometry using the methods of Mulvaney et al.^52^ and Miranda et al.^53^, respectively.

### Freeze-drying experimental design

The first experiment (FD-1) used six individual urine samples from three different sheep, ranging in pre-storage N concentrations from 1.3 to 11.9 g N l^−1^ (Supplementary Table 1). The selection of samples, with three from the same sheep, was made to check whether any differences in treatment might be due to differences between sheep. In addition, a gradient in concentration (covering the range of naturally observed urine concentrations at the site) was selected to explore possible trends in treatment response due to concentration over the wide range that has been reported in previous studies^16,23,43^. FD-1 investigated urine chemical composition and CO_2_ and N_2_O emissions from paired non-freeze-dried and freeze-dried urine samples.

The second experiment (FD-2) had six different mixed urine samples (with two individual urination events mixed together in each; Supplementary Table 1) to achieve two concentration levels (*ca*. 2 and 4 g N l^−1^) and investigated urine chemical composition and NO emissions from paired non-freeze-dried and freeze-dried samples. This enabled investigation of a concentration effect on the lyophilisation of urine and bulked urine samples are more representative of those usually applied in other experiments e.g.^11^.

### Freeze-drying experiments urine sample preparation and analysis

For each experiment, individual filtered (0.45 µm) and then stored frozen urine samples (*n* = 6 for FD-1 and *n* = 12 for FD-2) were defrosted in a refrigerator overnight (4 °C, 16 h) to enable preparation of the samples required. For FD-1, the six individual urine samples (Supplementary Table 1) were each divided in two subsamples (one subsample to remain non-freeze-dried and one for freeze-drying). For FD-2, the 12 individual urine samples were thoroughly mixed in pairs (Supplementary Table 1) and the resulting six mixed urine samples each divided in two subsamples (one subsample to remain non-freeze-dried and one for freeze-drying). The volume and mass of all subsamples was recorded. Subsamples to be freeze-dried were placed in over-sized plastic bottles, as urine samples could defrost slightly while the vacuum established and then initially frothed. The additional bottle headspace ensured this effervescence would be contained. All samples were then refrozen, initially at − 20 °C and then moved to − 80 °C. It was necessary to freeze samples at − 80 °C prior to freeze-drying due to the high solute concentration of urine to ensure they were completely frozen and did not defrost during placement in the freeze-dryer and vacuum establishment.

After freezing at − 80 °C at least overnight, the set of subsamples to be freeze-dried for each experiment were placed in the freeze-dryer, while the other set was moved back to storage at − 20 °C. Freeze-drying samples were monitored closely to remove approximately 80% of the water volume and ensure samples were not completely dried or left under vacuum for too long (freeze-drying for approximately 32 h was sufficient for the freeze-dryer and volumes used in this work). Freeze-drying to dryness was avoided to minimise the potential for loss of volatile urine constituents (e.g. NH_3_). Although not natural constituents, loss of volatile analytes (amphetamine and norpseudoephedrine) due to complete lyophilisation has been reported from urine samples evaluated for use as reference materials in doping studies^54^. In addition, partial freeze-drying reduces the likelihood of incomplete urine redissolution following freeze-drying observed elsewhere^39^. Freeze-dried urine samples were then rehydrated to their pre-freeze-drying volume and mass with ultrapure water and frozen at − 20 °C until required.

Non-freeze-dried and freeze-dried and rehydrated urine samples were diluted (1000-fold) with ultrapure water and analysed for total C and total N using a TOC-L total organic carbon analyser equipped with a TNM-L module (Shimadzu, Kyoto, Japan). Urine (undiluted) urea concentrations were determined by colorimetric reaction and spectrophotometry^55^. Urine (undiluted) NH_4_^+^-N and total oxidised nitrogen (TON) were analysed photometrically via modified versions of the Berthelot and Griess reactions, respectively, using an Aquakem 250 (Thermo Fisher Scientific Ltd.). Urine was diluted with ultrapure water as necessary to operate within the instrument working range to determine TFAAs by fluorescence^56^ and cations (Na^+^, K^+^ and Ca^2+^) by flame photometry. Urine TFAAs were measured in the mM range using a glycine standard and subsequently converted to mg l^−1^ using the molar mass of glycine (75.07 g mol^−1^) as glycine is the major amino acid present in urine^44^. Allantoin, creatinine, uric acid, hippuric acid and benzoic acid were analysed by high-performance liquid chromatography (HPLC) using a Varian Pro Star 310 HPLC System (Varian Inc., Palo Alto, CA). For HPLC analysis, urine samples were diluted with mobile phase A, as needed (10 to 50-fold), prior to analysis. Mobile phase A was monopotassium phosphate (KH_2_PO_4_; 17 g l^−1^; adjusted to pH 4) and mobile phase B comprised 60% mobile phase A and 40% HPLC-grade methanol (MeOH). The pumping rate was 1 ml min^−1^, through a C18 HyperClone 5 μm 12 nm ODS column (250 × 4.6 mm) column (Phenomenex Inc., Cheshire, UK). The UV detector wavelength was set at 218 nm.

### Soil sampling and characteristics for freeze-drying experiments

Soil for the gaseous emission measurements from non-freeze-dried and freeze-dried urine was randomly sampled (0–10 cm depth, *n* = 15 for FD-1 and *n* = 12 for FD-2) from the same semi-improved, extensively managed 11.5 ha pasture at the Henfaes Research Station from which sheep urine was collected. The pasture largely comprised a mosaic of bracken (*Pteridium aquilinum*; 60.2%) and semi-improved grassland (38.5%). The soil is classified as an Orthic Podzol^57^, but greater amounts of organic residues have built up beneath bracken stands. Individual samples were sieved (< 9 mm) to remove stones and plant roots and stored at 4 °C until required. The sieved soil samples (*n* = 15 for FD-1 and *n* = 12 for FD-2) were combined in equal dry weights to produce a representative pooled sample for each experiment.

### Freeze-drying urine-amended soil gaseous emission measurements

Measurement of (non-freeze-dried and freeze-dried) urine-amended soil gas emissions (CO_2_, N_2_O and NO) was conducted in the DENIS for both freeze-drying experiments. The DENIS allows emissions from 12 experimental units (vessels containing soil cores) to be determined under a controlled atmosphere (80:20 helium: oxygen; He:O_2_) per experiment. Each experimental unit was comprised of three 7.5 cm high, 4.5 cm in diameter repacked soil cores sealed inside a stainless-steel vessel.

In FD-1, soil cores were packed with pooled sieved soil (*n* = 15) to a bulk density of 0.8 g cm^−3^, while in FD-2, soil cores packed with pooled sieved soil (*n* = 12) had a bulk density of 0.7 g cm^−3^. Cores were packed in thirds (by depth) with tamping, compression to the correct density and surface roughening between each addition to ensure even bulk density throughout. Both bulk densities were representative of those found at the field site from which soil was collected. A high percentage water-filled pore space (% WFPS) of 80% was used in both experiments to facilitate denitrification and a relatively warm fixed incubation temperature of 18 °C was used throughout. This temperature was representative of a warm summer’s day at the field site from which the sheep urine and soil was obtained.

In each experiment, the repacked soil cores were flushed with 80:20 He:O_2_ at 30 ml min^−1^ for a 2-day pre-incubation period to allow the cores to settle. Flows were then adjusted over the surfaces of the cores to 12 ml min^−1^ for 24 h prior to urine application. Urine samples were defrosted in a refrigerator overnight and allowed to reach room temperature prior to application. Urine (5 ml per core by injection onto core surface) was applied to each vessel in sequence with the gas chromatograph runs (each lasting 8 min), such that urine was applied to a vessel 8 min before gas from that vessel was sent to the gas chromatograph. In both experiments, urine treatments were randomised between vessels, but paired samples were adjacent in the sampling sequence to minimise differences between them (Supplementary Fig. 1a,b).

Soil CO_2_ and N_2_O emissions were determined using a Pye Unicam 4500 gas chromatograph (Philips Scientific, Unicam Ltd., Cambridge, UK) fitted with an electron capture detector. The carrier gas was ECD-grade nitrogen, flowing at 0.6 kg cm^−2^ through a 2 m stainless steel Porapak Q packed column (i.d. 4 mm, 80–100 mesh). The injector temperature was set at 100 °C, the oven maintained at 60 °C and the detector operated at 250 °C. The purge gas was ECD-grade nitrogen, flowing at 0.4 kg cm^−2^. Soil NO emissions were analysed using a Sievers Nitric Oxide Analyser 280i (GE Water and Process Technologies Analytical Instruments, Boulder, Colorado, USA). The instrument measures NO through the gas phase chemiluminescent reaction between NO and ozone (generated from an O_2_ supply) using a photomultiplier tube detector.

### Data processing and statistical analysis

The data from the urine storage test were analysed using linear mixed models (LMMs) to assess the effects of filtration and storage temperature on sheep urine N constituents over time. The fixed model used was filter*temp*time and the random model was sample/time. An antedependence order one correlation structure was used to account for possible correlation between repeated measurements taken on the same units over time. The NH_4_^+^-N and NO_3_^–^N data were natural logarithm (LN) transformed.

Analysis of variance (ANOVA) with blocking to account for paired non-freeze-dried (N) and freeze-dried (F) samples was used to assess whether urine constituent concentrations were significantly different with and without freeze-drying (and for FD-2, whether this was different with concentration using a crossed treatment structure). Some variables required transformation to better satisfy the requirements for normality and constant variance as follows: for FD-1, total C, K^+^ and creatinine were square root (SQRT) transformed, while NH_4_^+^-N, TFAAs, Na^+^, Ca^2+^, allantoin, uric acid, hippuric acid and benzoic acid were LN transformed; for FD-2, urea-N, K^+^ and creatinine were SQRT transformed, while total C, total N, TFAAs, allantoin, uric acid and hippuric acid were LN transformed.

Cumulative CO_2_-C, N_2_O-N and NO-N were calculated using the area under the curve of hourly fluxes. Where necessary (for CO_2_-C and N_2_O-N), hourly fluxes were baseline corrected (on an individual vessel basis) by subtraction of mean pre-treatment fluxes. Analysis of variance was also used to assess whether emissions of cumulative CO_2_-C, N_2_O-N and NO-N from soil amended with non-freeze-dried and freeze-dried sheep urine were significantly different (and for FD-2, whether this varied with concentration). Cumulative CO_2_-C emissions were LN transformed. Cumulative N_2_O-N and cumulative NO-N emissions were SQRT transformed. Genstat (19th Edition; VSNi) was used for statistical analyses.

### Ethics approval

For sheep urine collection—Bangor University’s College of Natural Sciences Ethics Committee; code: CNS2016DC01.

## Supplementary Information

Supplementary Information.

## Data Availability

The datasets generated during this study will be made available in the NERC Environmental Information Data Centre (EIDC) which is hosted by the UK Centre for Ecology & Hydrology (UKCEH). In the interim, data are available from the corresponding author on reasonable request.
