# Supplementary material for: Optimising storage conditions and processing of sheep urine for nitrogen cycle and gaseous emission measurements from urine patches
Source: Sci Rep. 2021 Jun 9;11:12116. doi: 10.1038/s41598-021-91498-4 (PMC8190061; doi:10.1038/s41598-021-91498-4)
Supplement: Supplementary file 1 — Supplementary Information. [file 41598_2021_91498_MOESM1_ESM.docx]

**Optimising storage conditions and processing of sheep urine prior to subsequent nitrogen-cycle and gaseous emission measurements from urine patches**

Alice F. Charteris^1^, Karina A. Marsden^2,3^, Jess R. Evans^4^, Harry A. Barrat^1^, Nadine Loick^1^, Davey L. Jones^2,5^, David R. Chadwick^2^ and Laura M. Cárdenas^1*^

^1^*Sustainable Agriculture Sciences, Rothamsted Research, North Wyke, Okehampton, Devon, EX20 2SB, UK*

^2^*School of Natural Sciences, Bangor University, Bangor, Gwynedd, LL57 2UW, UK*

*^3^Faculty of Veterinary and Agricultural Sciences, University of Melbourne, Parkville, Victoria 3010, Australia*

^4^*Rothamsted Research, Harpenden, Hertfordshire, AL5 2JQ, UK*

^5^*SoilsWest, UWA School of Agriculture and Environment, The University of Western Australia, Perth, WA 6009, Australia*

*Corresponding author: Laura M. Cárdenas - laura.cardenas@rothamsted.ac.uk

**Supplementary Information**

Supplementary Table 1. Sheep urine samples used in the composition and gas emission experiments. Samples in FD-1 are identified by number in order of increasing urine total N concentration. Samples in FD-2 are identified by ‘LM’ for ‘low mixture’ or ‘HM’ for ‘high mixture’ and numbered in order of increasing total N concentration. Sheep urine sample numbers indicate the sheep from which the urine was collected and the urination event (thus 6.2 is sheep six, second urination event).

|  | Sample ID | Sheep urine sample | Total C /g l^-1^ | Total N /g l^-1^ |
| --- | --- | --- | --- | --- |
| FD-1 | 1 | 1.1 | 2.80 | 1.28 |
|  | 2 | 3.2 | 7.19 | 3.00 |
|  | 3 | 4.18 | 10.66 | 5.91 |
|  | 4 | 6.2 | 14.29 | 7.20 |
|  | 5 | 3.1 | 18.54 | 9.35 |
|  | 6 | 3.6 | 26.30 | 11.93 |
| FD-2 | LM1 | 4.16+4.6 | 3.88 | 1.87 |
|  | LM2 | 4.10+1.1 | 5.17 | 2.05 |
|  | LM3 | 4.3+4.11 | 3.63 | 2.10 |
|  | HM4 | 4.14+4.1 | 6.93 | 3.79 |
|  | HM5 | 4.8+4.4 | 10.06 | 3.87 |
|  | HM6 | 4.5+5.1 | 9.04 | 4.15 |

Supplementary Table 2. Urine storage tests N constituent LMM results (F statistics, numerator degrees of freedom [n.d.f.], denominator degrees of freedom [d.d.f.] and probabilities). Stars highlight significant results (* p<0.05, ** p<0.01, *** p<0.001).

|  |  | Filtering | Temperature | Time | Filtering*Temperature | Filtering*Time | Temperature*Time | Filtering*Temperature*Time |
| --- | --- | --- | --- | --- | --- | --- | --- | --- |
| Total N | F | 4.31 | 48.80 | 100.58 | 1.29 | 4.58 | 14.22 | 6.04 |
|  | n.d.f. | 1 | 2 | 4 | 2 | 4 | 8 | 8 |
|  | d.d.f. | 11.9 | 11.9 | 13.9 | 11.9 | 13.9 | 17.0 | 17.0 |
|  | p | 0.060 | <0.001*** | <0.001*** | 0.312 | 0.014* | <0.001*** | <0.001*** |
| LN(NH_4_^+^-N) | F | 6025.20 | 60451.17 | 50918.52 | 2614.00 | 483.56 | 8143.78 | 595.18 |
|  | n.d.f. | 1 | 2 | 4 | 2 | 4 | 8 | 8 |
|  | d.d.f. | 11.6 | 11.6 | 12.8 | 11.6 | 12.8 | 15.7 | 15.7 |
|  | p | <0.001*** | <0.001*** | <0.001*** | <0.001*** | <0.001*** | <0.001*** | <0.001*** |
| LN(NO_3_^-^-N) | F | 0.82 | 13.64 | 97.54 | 0.63 | 15.94 | 21.89 | 6.33 |
|  | n.d.f. | 1 | 2 | 4 | 2 | 4 | 8 | 8 |
|  | d.d.f. | 11.8 | 11.8 | 13.1 | 11.8 | 13.1 | 16.0 | 16.0 |
|  | p | 0.382 | <0.001*** | <0.001*** | 0.548 | <0.001*** | <0.001*** | <0.001*** |
| Total organic N | F | 2918.50 | 14523.88 | 437.56 | 1396.36 | 32.32 | 257.53 | 145.41 |
|  | n.d.f. | 1 | 2 | 4 | 2 | 4 | 8 | 8 |
|  | d.d.f. | 11.9 | 11.9 | 14.4 | 11.9 | 14.4 | 17.5 | 17.5 |
|  | p | <0.001*** | <0.001*** | <0.001*** | <0.001*** | <0.001*** | <0.001*** | <0.001*** |

Supplementary Table 3. FD-1 and FD-2 ANOVA results (F statistics and probabilities) for urine constituent concentrations without (N) and with (F) freeze-drying. For both experiments, one degree of freedom was associated with the treatment and for FD-1 there were five residual degrees of freedom, whereas for FD-2, there were four. Stars highlight significant results (* p<0.05, ** p<0.01, *** p<0.001). Italicised p values are close to significance at the 5% level.

|  |  | FD-1 | FD-2 | | |
| --- | --- | --- | --- | --- | --- |
|  |  | N/F | Conc. | N/F | N/F^*^Conc. |
| Total C ^a, d^ | F | 0.11 | 19.98 | 1.86 | 5.15 |
|  | p | 0.758 | 0.011* | 0.244 | 0.086 |
| Total N ^d^ | F | 1.33 | 246.38 | 0.19 | 1.51 |
|  | p | 0.301 | <0.001*** | 0.688 | 0.286 |
| NH_4_^+^-N ^b^ | F | 9.75 | 2.17 | 6.34 | 1.8 |
|  | p | 0.026* | 0.214 | 0.066 | 0.251 |
| TON-N | F | - | 3.65 | 0.06 | 0.06 |
|  | p | - | 0.129 | 0.812 | 0.812 |
| Urea-N ^c^ | F | 0 | 60.78 | 13.25 | 9.34 |
|  | p | 0.992 | 0.001** | 0.022* | 0.038* |
| TFAAs ^b, d^ | F | 2.09 | 1.84 | 0.34 | 2.46 |
|  | p | 0.208 | 0.247 | 0.591 | 0.192 |
| Na^+ b^ | F | 0.18 | 0.42 | 0.03 | 3.71 |
|  | p | 0.691 | 0.553 | 0.873 | 0.126 |
| K^+ a, c^ | F | 0.21 | 7.18 | 6.94 | 9.37 |
|  | p | 0.667 | *0.055* | *0.058* | 0.038* |
| Ca^2+ b^ | F | 2.27 | 4.66 | 0.21 | 0.14 |
|  | p | 0.192 | 0.097 | 0.669 | 0.723 |
| Allantoin ^b, d^ | F | 1.51 | 14.89 | 0.07 | 1.73 |
|  | p | 0.725 | 0.018* | 0.806 | 0.259 |
| Creatinine ^a, c^ | F | 0.82 | 6.76 | 0.11 | 0.92 |
|  | p | 0.406 | 0.06 | 0.756 | 0.391 |
| Uric acid ^b, d^ | F | 2.48 | 44.04 | 0.3 | 0.06 |
|  | p | 0.176 | 0.003** | 0.612 | 0.821 |
| Hippuric acid ^b, d^ | F | 1.7 | 8.2 | 1.28 | 2.53 |
|  | p | 0.249 | 0.046* | 0.321 | 0.187 |
| Benzoic acid ^b^ | F | 0.02 | 8.46 | 0.69 | 1.16 |
|  | p | 0.907 | 0.044* | 0.454 | 0.341 |

^a^ SQRT transformed in FD-1.

^b^ LN transformed in FD-1.

^c^ SQRT transformed in FD-2.

^d^ LN transformed in FD-2.

a

3-N

1-F

5-N

1-N

3-F

6-N

4-F

2-F

2-N

6-F

4-N

5-F

1

2

7

6

5

4

3

8

9

11

10

12

b

LM2-N

HM5-N

LM3-N

HM5-F

LM2-F

HM4-N

LM1-N

HM6-F

HM6-N

HM4-F

LM1-F

LM3-F

1

2

7

6

5

4

3

8

9

11

10

12

**Supplementary Figure 1** DENIS urine treatment allocation for: (a) FD-1 and (b) FD-2. Numbers outside circles represent DENIS vessel number and gas sampling sequence. Numbers within circles are sheep urine sample IDs from Supplementary Table 1 and ‘N’ if for non-freeze-dried, while ‘F’ is freeze-dried.
